# Waist-to-height ratio as a non-invasive marker of renal sinus fat: a MRI-based cohort study

**DOI:** 10.1038/s41366-025-01974-4

**Published:** 2025-12-13

**Authors:** Diego Moriconi, Miikka-Juhani Honka, Ekaterina Saukko, Emilia Moritz, Aino Latva-Rasku, Prince Dadson, Nelli Tuomola, Laura Pekkarinen, Paulina Salminen, Pirjo Nuutila, Eleni Rebelos

**Affiliations:** 1 Department of Clinical and Experimental Medicine, University of Pisa, Pisa, Italy; 2 Turku PET Centre, University of Turku, Turku, Finland; 3 Institute of Clinical Physiology, National Research Council (CNR), Pisa, Italy; 4 Department of Radiology, Turku University Hospital, Turku, Finland; 5 Department of Endocrinology, Turku University Hospital, Turku, Finland; 6 Division of Digestive Surgery and Urology, Turku University Hospital, Turku, Finland; 7 InFLAMES Research Flagship, University of Turku, Turku, Finland; 8 Diabetes Center, First Department of Propaedeutic and Internal Medicine, Medical School, National and Kapodistrian University of Athens, Laiko General Hospital, Athens, Greece

**Keywords:** Clinical trial design, Metabolism

## Abstract

**Background and aims:**

Renal sinus fat (RSF) is an ectopic fat depot whose expansion has been linked to hypertension and chronic kidney disease. We assessed a range of adiposity indices to determine whether they offer more accurate predictions of RSF than BMI.

**Methods and results:**

Renal sinus fat (RSF) and RSF relative to total kidney area (RSF%) were assessed via MRI in 74 individuals with severe obesity and 47 lean volunteers. 50 persons with obesity were re-evaluated 6 to 12 months after undergoing bariatric surgery. In multivariable regression analyses adjusted for age, sex, and BMI, the Body Roundness Index (BRI), waist-to-height ratio (WHtR), and waist circumference showed the strongest associations with RSF. Of these, only WHtR was significantly associated with RSF%. In univariate analyses, both RSF and RSF% were inversely correlated with estimated glomerular filtration rate (eGFR); however, in multivariate analysis, only RSF% remained independently associated with eGFR. Post-bariatric surgery, RSF change correlated with changes in WHtR and BRI.

**Conclusion:**

Adiposity measures incorporating waist circumference are associated with RSF independent of BMI. While RSF exhibits a stronger relationship with adiposity measures, RSF% predicts eGFR. Both metrics offer complementary insights and should be considered in future studies.

## Introduction

The global rise in obesity has highlighted the need for accurate, accessible, and clinically relevant anthropometric indices capable of assessing visceral fat accumulation and related cardiometabolic risks [1]. Conventional measures of adiposity, such as body mass index (BMI) fail to distinguish between lean and fat mass and cannot capture regional adipose tissue distribution [2]. In contrast, indices that incorporate waist circumference, such as waist-to-height ratio (WHtR), Body Roundness Index (BRI), and the Visceral Adiposity Index (VAI), have been proposed as more reliable indicators of central adiposity and metabolic dysfunction [3–5].

Recent evidence indicates that central obesity is strongly associated with ectopic fat deposition in organs such as the liver, pancreas and also in the region of the kidneys [6, 7]. Renal sinus fat (RSF) is an ectopic adipose depot located in the renal hilum (sinus renalis), surrounding the renal vasculature and collecting system rather than within the renal parenchyma. Accumulation of RSF is thought to contribute to intrarenal pressure, activation of the renin–angiotensin–aldosterone system, and impaired renal perfusion, potentially leading to hypertension and chronic kidney disease [8, 9].

Although RSF can be quantified using imaging techniques such as magnetic resonance imaging (MRI) or computerized tomography (CT) [10], these methods are not routinely available in clinical practice. Therefore, the ability to estimate RSF through non-invasive anthropometric indices may represent a practical approach to identify individuals at risk. However, few studies have directly assessed whether commonly used adiposity measures, are associated with RSF. Previous studies, such as the Framingham Heart Study, have assessed the relationship between RSF and general or central adiposity indices. However, these analyses focused exclusively on absolute RSF, without accounting for the kidney size. More recently, Fujioka et al. proposed the use of renal sinus fat percentage (RSF%), defined as RSF volume relative to total kidney volume, suggesting that the proportional fat burden may be more functionally relevant than absolute RSF volume [11].

In this context, the present study aimed to investigate the relationship between various adiposity indices and both single-slice RSF area and RSF% (i.e. RSF area to total kidney area, measured from the same slice), as well as to explore whether RSF% is associated with renal function. Specifically, we sought to determine whether non-invasive measures of central adiposity could predict MRI-derived RSF or RSF%.

## Methods

### Participants and study design

We used the dataset that we have previously compiled where we assessed the association between RSF and hypertension status [9]. In brief, data from three clinical studies were analyzed. The final dataset comprised 74 persons with severe obesity and 47 healthy lean volunteers. Persons with obesity who were referred to the Turku University Hospital for bariatric surgery were recruited. Healthy lean volunteers were recruited via an advertisement in local newspapers. The inclusion and exclusion criteria have been previously described [12, 13]. In brief, inclusion criteria were age 18–65 years of age and for the subjects with obesity BMI > 40 kg/m^2^ or > 35 kg/m^2^ with an additional risk factor. Individuals using insulin treatment and/or with mental disorders, eating disorders, or poor compliance were excluded, as were those with a body weight over 150 kg, because of restrictions of the imaging devices. Subjects were also eligible to undergo MRI. 50 persons with severe obesity were re-studied 6 months to 1 year following bariatric surgery. In that occasion, anthropometric, laboratory and MRI characteristics were re-assessed. The study protocols were approved by the Ethics Committee of the Hospital District of Southwest Finland, and all subjects gave their written informed consent before participating in the study (NCT00793143; studies performed from March 2009 to October 2010, NCT01373892; studies performed from March 2011 to October 2013 and NCT04343469; studies performed from February 2019 to June 2021).

### Clinical assessments

All subjects underwent an initial screening visit after an overnight fast, during which a detailed medical history was recorded. Blood pressure was measured with OMRON711 automatic blood pressure monitor (Omron Corporate, Kyoto, Japan). Before the measurements, subjects were sitting for >10 min in a quiet room. A study nurse then assessed each subject twice for blood pressure measurements within a five-minute interval, and the average value was considered for the analysis. Fasting blood and urine samples were collected and then a standard 75-gr oral glucose tolerance test (OGTT) with frequent blood sampling every 30 minutes was performed for measurement of plasma glucose, insulin and C-peptide.

On a separate day, subjects underwent whole-body magnetic resonance imaging (MRI) with either a Philips Gyroscan Intera 1.5 T CV Nova Dual scanner (Philips, Amsterdam, The Netherlands) or with a Siemens Magnetom Skyra fit 3T MRI scanner (Siemens Medical Solutions, Erlangen, Germany). MRI acquisition was performed with axial T1-weighted dual fast field echo images (echo time (TE) 2.3 and 4.6 ms, repetition time (TR) 120 ms, slice thickness 10 mm without gap, matrix 256 256) or with T1-weighted 3D VIBE two-point DIXON sequence in breath-hold mode (TE 1.2 and 2.5 ms, TR 4.0 ms, slice thickness 2 mm with 0.4 mm gap, matrix 182 × 224. Subjects were scanned from head to knee or to ankle in a supine position. Total scan duration was 20 min.

### RSF assessment

**Fig. 1:**
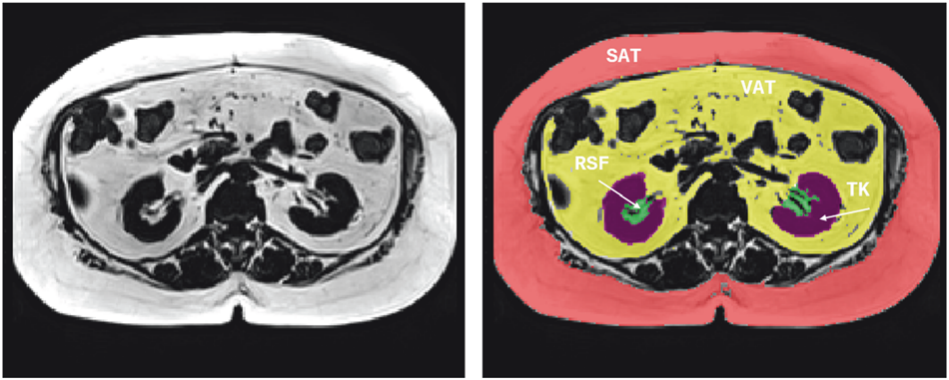
Example of region of interest placement; Arrows indicate renal sinus fat and total kidney area.

RSF determination was done on a single MRI slice using the SliceOmatic Tomovision software (version 4.3), as previously described [9]. In brief, after visual inspection of the whole kidney area, the areas of renal sinus fat on both kidneys were identified by using anatomic landmarks and were manually segmented within the curvature of the kidney, excluding the renal vasculature and the renal collecting system. Left and right RSF were averaged and used in the analyses. RSF analyses were performed blindly by two operators (E.M. and E.S.); the interrater variability and ICC estimate of the RSF measurement have been previously reported, suggesting good to excellent agreement [9]. Total area of the kidney from the same single-slice used to assess RSF (without including RSF) was also assessed using the SliceOmatic Tomovision software (version 4.3). RSF% was calculated as (RSF/total kidney area)*100, and expresses the % of renal sinus fat compared to the total area of the kidney. An example of region of interest placement is shown in Fig. 1.

### Adiposity indices

Waist-to-height ratio (WHtR) was calculated as waist circumference divided by height. The Body Roundness Index (BRI) and A Body Shape Index (ABSI) were computed using published formulas incorporating waist circumference, height, and weight, as previously described [14]; BRI = 364.2–5.5* √(1 − (waist circumference/2*π*)^2^/(0.5 × height)^2^) and ABSI= waist circumference/(BMI^2/3^*height^1/2^).

The VAI was calculated based on waist circumference, BMI, triglycerides, and HDL cholesterol, using sex-specific equations [15].

### Other calculations

Oral glucose insulin sensitivity (OGIS) was calculated from the OGTT data as previously described [16]. Estimated glomerular filtration rate (eGFR) was calculated by the Chronic Kidney Disease Epidemiology Collaboration (CKD-EPI) equation [17].

### Statistical analysis

Continuous variables are summarized as mean ± SD, or as median [interquartile range]. The normality of distribution was assessed using the Shapiro-Wilk test. Between groups comparisons were performed by *t*-test or Mann-Whitney U test, as appropriate.

Univariate associations between RSF, RSF%, eGFR, OGIS, and adiposity indices (BMI, BRI, waist circumference, WHtR, VAI, ABSI) were explored using Pearson correlation coefficients.

Multivariate linear regression models were then constructed to assess the independent associations between RSF and adiposity indices, and between OGIS (as a marker of insulin sensitivity) or eGFR (as a marker of renal function) and RSF, RSF%, or anthropometric indices. All models were adjusted for potential confounding variables, including age, sex, and mean arterial pressure or BMI. Each regression model included one adiposity index at the time to avoid multicollinearity. Standardized beta coefficients (st.β) were reported to facilitate the comparison of effect sizes across variables with different units. Multicollinearity was assessed by calculating the variance inflation factor (VIF) for each predictor. ANOVA per repeated measures was performed to assess differences in BMI, and eGFR following bariatric surgery. Statistical analyses were done using JMP version 17.0 (SAS Institute, Cary, NC, USA). A *p* value < 0.05 was considered statistically significant.

## Results

### Clinical and metabolic characteristics of study participants

As shown in Table 1, individuals affected by obesity had, by definition, significantly higher BMI, waist circumference, BRI and waist-to-height values compared to normal-weight controls. Systolic and diastolic blood pressures were also elevated in the BMI ≥ 30 group, as were fasting glucose, HbA_1c_, and insulin. Consequently, insulin sensitivity, measured using OGIS, was significantly reduced in individuals affected by obesity. Similarly, C-reactive protein levels, a marker of systemic inflammation, were elevated in this group. There were no differences in eGFR between the two groups, and across all subjects eGFR ranged from 60 to 140 ml/min/1.73 m^2^.

**Table 1:** Anthropometrics and clinical features of subjects affected by obesity vs healthy lean controls.

	People with obesity	Lean controls	*p* value
*n*	74	47	
Age (years)	45 ± 10	46 ± 10	0.618
Sex (f/m)	69/5	36/11	0.033
BMI (kg/m^2^)	41.8 ± 4.1	23.7 ± 2.5	<0.001
Waist circumference (cm)	118 ± 10	80 ± 9	<0.001
BRI	8.25 ± 1.78	2.95 ± 0.95	<0.001
VAI	2.05 ± 1.09	1.04 ± 0.77	<0.001
ABSI	7.65 ± 0.57	7.46 ± 0.55	0.023
WHtR	0.71 ± 0.06	0.47 ± 0.05	<0.001
Total VAT (kg)	4.68 ± 1.97	1.77 ± 1.33	<0.001
SBP (mmHg)	134 ± 17	125 ± 13	0.005
DBP (mmHg)	86 ± 10	79 ± 8	<0.001
eGFR (ml/min/1.73 m^2^) GFR (ml/min/1.73 m^2^)	100 ± 14	97 ± 13	0.274
Fasting glucose (mmol/L)	6.0 ± 0.9	5.2 ± 0.5	<0.001
HbA_1c_ (mmol/mol)	39.9 ± 6.2	35.6 ± 4.1	0.003
Insulin (pmol/L)	92 ± 55	35 ± 20	<0.001
OGIS (ml/min^*^m^−2^)	337 ± 52	426 ± 56	<0.001
C-Reactive Protein (mg/dL)	3.2 [1.7–5.3]	0.6 [0.2–1.0]	<0.001
RSF (cm^2^)	2.47 ± 1.08	1.99 ± 0.79	0.018
RSF (%)	11.8 ± 4.8	10.7 ± 4.3	0.355

### Associations between adiposity indices and RSF

RSF was significantly higher in the group with obesity compared to lean subjects (2.47 ± 1.08 vs. 1.99 ± 0.79 cm², *p* = 0.018) (Table 1). In univariate analysis, RSF was directly related to age (*r* = 0.29, *p* < 0.001), BMI (*r* = 0.26, *p* = 0.002) and with all the four estimate measures of adiposity, WC (*r* = 0.40, *p* < 0.001), BRI (*r* = 0.37, *p* < 0.001), ABSI (*r* = 0.38, *p* < 0.001) and VAI (*r* = 0.30, *p* < 0.001).

Multivariate linear regression models were used to assess the independent associations between adiposity indices and RSF, after adjusting for age, sex, and BMI (Table 2). BRI (st.*β* = 0.50, *p* = 0.017), WHtR (st.*β* = 0.54, *p* = 0.016) and waist circumference (st.*β* = 0.55, *p* = 0.012) demonstrated the strongest associations with RSF. ABSI showed a weaker but still statistically significant association (st.*β* = 0.18, *p* = 0.035), whereas VAI did not reach statistical significance (st.*β* = 0.17, *p* = 0.055). RSF was also inversely related to OGIS (*r* = −0.25, *p* = 0.01), but this association did not remain significant after accounting for BMI.

**Table 2:** Adjusted *R*² and standardized beta coefficients (β) from linear regression models evaluating the relationship between **A**) RSF, **B**) RSF% and body adiposity indices, adjusted for age, sex, and BMI.

A)RSF	Adjusted R^2^	St.β	*p* value
BRI	0.33	0.50	0.017
WHtR	0.33	0.54	0.016
VAI	0.32	0.17	0.055
ABSI	0.32	0.18	0.035
Waist circumference (cm)	0.33	0.55	0.012
**A)RSF%**	**Adjusted R**^**2**^	**St.β**	***p*** **value**
BRI	0.16	0.44	0.061
WHtR	0.16	0.52	0.038
VAI	0.15	0.08	0.396
ABSI	0.13	0.16	0.086
Waist circumference (cm)	0.15	0.40	0.102

**Fig. 2:**
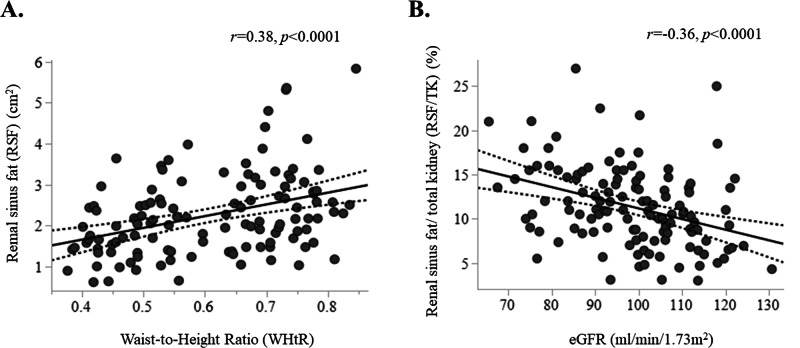
Correlations of renal sinus fat with clinical parameters. Renal sinus fat was directly associated with Waist-to-Height ratio (WHtR) (**A**). Renal sinus fat percentage (RSF%) was inversely associated with estimated glomerular filtration rate (eGFR) (**B**). The dotted lines mark the 95% confidence interval.

Conversely, there were no differences in RSF% in the two groups. In univariate analysis, RSF% was directly related to age (*r* = 0.28, *p* = 0.001) and ABSI (*r* = 0.27, *p* = 0.001). RSF% had a weaker association with WHtR (*r* = 0.16, *p* = 0.050) but was not associated with BMI (*p* = 0.59), other adiposity measures, or OGIS. In multivariable regression analysis, when RSF% was used as the dependent variable, only WHtR showed a significant positive association (st.*β* = 0.52, *p* = 0.038) (Fig. 2A), whereas none of the other adiposity indices were significantly associated with RSF% (Table 2). However, variance inflation factors (VIF) between BMI and other adiposity indices resulted >5 in several models, indicating high collinearity. Therefore, additional models were run excluding BMI. In this context, using only age and sex as independent variable, also ABSI (st.*β* = 0.18, *p* = 0.047) and BRI (st.*β* = 0.16, *p* = 0.050) became independently associated with RSF%, suggesting that collinearity with BMI may have obscured their contribution in the full model.

### Association between adiposity index, RSF and renal function

In univariate analysis, eGFR was not significantly associated with BMI, BRI, waist circumference, VAI or WHtR. In contrast, both RSF and RSF% demonstrated significant inverse correlations with eGFR (*r* = 0.20, *p* = 0.014 and *r* = 0.35, *p* < 0.001, respectively).

In multivariate models adjusting for age, sex, and mean blood pressure, the association between RSF and eGFR was no longer significant (*p* = 0.296), whereas RSF% remained independently associated with eGFR (st.β = -0.27, *p* = 0.002) (Fig. 2B).

Since RSF may represent a local expression of visceral adiposity, we also examined whether visceral adipose tissue (VAT) was associated with kidney function. In univariate analyses, VAT was not significantly related to eGFR (*p* = 0.82).

### Change in RSF following weight loss associated with a change in WHtR

**Fig. 3:**
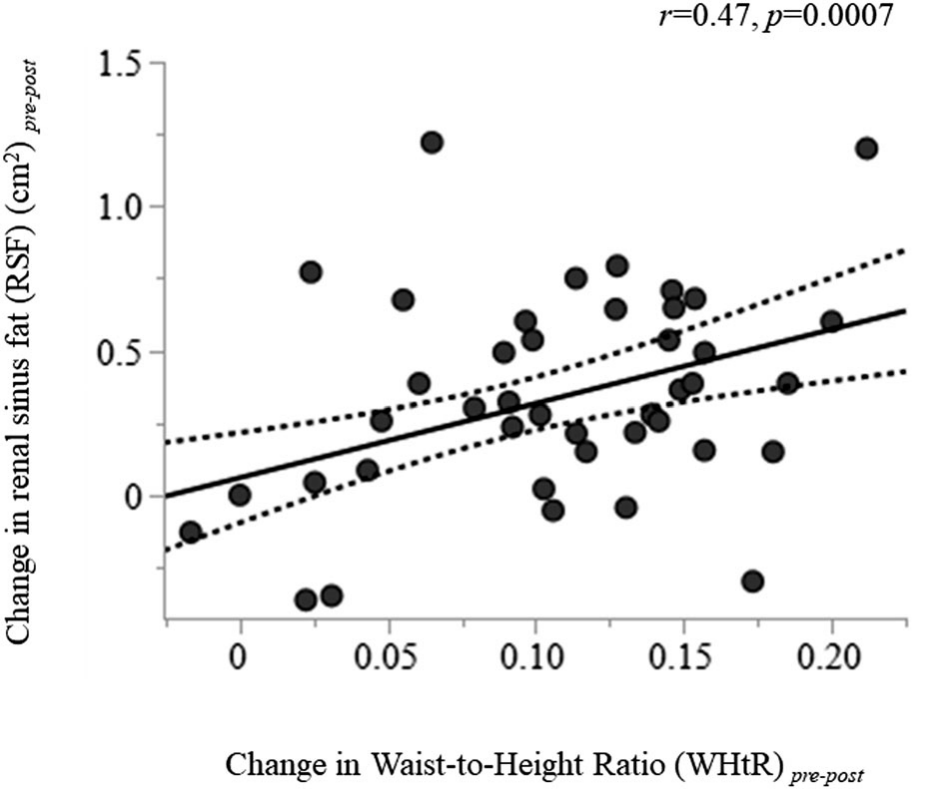
Change in renal sinus fat was directly associated with change in waist-to-height ratio (WHtR). The dotted lines mark the 95% confidence interval.

50 persons with obesity were re-studied following bariatric surgery (from 6 months to 1 year following intervention). On average, they had lost 9.7 ± 2.8 units of BMI, whereas eGFR was increased (102 ± 13 *vs* 119 ± 25 ml/min/1.73 m^2^, *p* < 0.0001). We found that change in RSF was associated with change in WHtR (*r* = 0.47, *p* = 0.0007) (Fig. 3), and with change in BRI (*r* = 0.28, *p* = 0.047), whereas there was no association with change in BMI, change in ABSI, or change in waist circumference.

Additionally, no association was observed between change in RSF% and change in any of the adiposity measures.

### Sensitivity analysis

Because of the pronounced female predominance in the group with obesity, we conducted a sensitivity analysis restricted to women only, to ensure that the observed associations were not driven by sex imbalance. The associations between WHtR and RSF and between WHtR and RSF% were preserved (st.*β* = 0.68, *p* = 0.008 and st.*β* = 0.71, *p* = 0.001, respectively). Moreover, RSF% also maintained a significant inverse correlation with eGFR (CKD-EPI) (*r* = –0.38, *p* < 0.001), confirming the robustness of our findings.

## Discussion

In this study, we evaluated the relationship between several anthropometric adiposity indices and RSF, considering both its absolute area and its proportion relative to kidney size (RSF%). We also assessed whether RSF, or RSF% were independently associated with renal function, as estimated by eGFR.

First, we found that multiple adiposity indexes – including BRI, WHtR, waist circumference, and ABSI- were associated with RSF, independently of age, sex and BMI. Of note, when each one of these adiposity measures was included in the same statistical model with BMI, BMI was no longer a significant predictor of RSF. Thus, these adiposity measures that account for waist circumference outperform BMI for the prediction of RSF. On the contrary, among the adiposity indices tested, only WHtR exhibited an independent association with RSF%. The stronger association of WHtR with RSF% compared with waist circumference or BRI likely reflects its adjustment for body height. Waist circumference alone does not distinguish between shorter and taller individuals, whereas WHtR normalizes abdominal size to stature, thereby capturing central adiposity relative to body frame. This is consistent with prior evidence that WHtR outperforms BMI and WC in predicting visceral and ectopic fat depots [18, 19].

Taken together, these findings suggest that indices reflecting central fat distribution, such as WHtR are more closely linked to RSF accumulation than BMI or total adiposity. This is consistent with prior evidence indicating that WHtR and ABSI outperforms BMI in predicting visceral fat and cardiometabolic risk [18, 20]. Here of all the adiposity indexes assessed the one that showed the most consistent results was WHtR. This was also confirmed in the smaller set of persons who underwent bariatric surgery and were re-studied following weight loss. Change in WHtR closely associated with change in RSF; moreover this finding underscores that following a weight loss intervention, RSF is also decreased, which may have beneficial effects for renal function [21].

Second finding of the present study was that RSF% (i.e. RSF corrected to kidney volume) but not absolute RSF volume, was independently and inversely associated with eGFR, even after adjusting for covariates such as age, sex, and blood pressure. This finding suggests that the proportion of renal sinus fat relative to kidney volume rather than fat volume alone may be a more sensitive marker of kidney stress, a hypothesis also supported by Fujioka et al. in patients with CKD [11]. Our study expands on the previous findings from Fujioka et al., as in the present study participants had normal renal function, with eGFR ranging from 60 to 140 ml/min/1.73 m^2^. This interval is conventionally considered normal, although values in the upper range may actually represent renal hyperfiltration in some subjects, a phenomenon common in obesity and associated with early kidney injury.

As such, we think that RSF% may represent an important index to study early insult to the kidney in the context of obesity, and insulin resistance; besides it is well-established that obesity represents an independent risk factor for progression to CKD [22]. On the other hand, the distinct pattern of associations between RSF and RSF% suggests that future studies utilizing MRI or CT kidney data should report both RSF and RSF%, as these parameters appear to provide complementary insights. RSF is more closely linked to adiposity, whereas RSF% reflects renal function, as estimated by eGFR.

From a mechanistic standpoint, RSF may contribute to renal dysfunction through both hemodynamic and endocrine pathways [23–25]. Accumulation of fat in the renal sinus may compress intrarenal vessels and lymphatics, raise intrarenal pressure, and promote hypoxia, all of which can impair glomerular filtration [26, 27]. Moreover, RSF has been linked to the activation of the renin–angiotensin–aldosterone system, further exacerbating hypertension and renal injury [28, 29]. Beyond these hemodynamic effects, recent transcriptomic analyses suggest that RSF may also act as an inflammatory adipose depot, with upregulation of immune-related pathways such as IL-17, TNF-α, and NF-κB compared with subcutaneous fat [30]. This inflammatory profile may further contribute to renal injury, supporting the hypothesis that RSF is not merely an anatomical bystander but a metabolically and immunologically active tissue.

Clinically, our results suggest that WHtR could serve as a simple anthropometric marker to identify individuals with disproportionately high RSF accumulation, particularly those at risk for early renal dysfunction. Our findings are consistent with recent population-based cohorts, including individuals with obesity, normal weight, and older adults, in which WHtR, VAI, and other adiposity indices have shown stronger associations with CKD risk than BMI alone [19, 31, 32].

Since RSF is not routinely measured in clinical practice and advanced imaging is not universally accessible, simple anthropometric indices such as WHtR may provide practical surrogates to estimate ectopic renal fat burden and help stratify patients with obesity who are particularly prone to CKD. In our cohort, WHtR was strongly associated with RSF and importantly, eGFR was related to RSF but not to VAT, suggesting that RSF and, indirectly, its surrogate WHtR, capture pathophysiological features relevant for kidney damage. These observations remain exploratory and should be regarded as hypothesis-generating rather than practice-changing, requiring confirmation in larger and longitudinal cohorts across different stages of kidney function.

Strengths of the present investigation are the application of MRI to assess RSF, the assessment in the same report of both RSF and RSF%, and the inclusion of subjects with normal renal function at risk for developing CKD in the future due to severe obesity. Healthy lean controls matched for age and sex were also studied, in order to compare group differences, in a cross-sectional manner. Some limitations of the present study should be acknowledged. Only 50 persons with obesity returned for the post-bariatric surgery evaluation, whereas lean subjects were studied only once. Moreover, the relatively short follow-up of 6–12 months only precludes causal inferences between RSF accumulation and decline in eGFR in people with obesity. Additionally, anthropometric indices were evaluated independently in separate models to avoid collinearity, but this may underestimate potential synergistic effects. Concerning kidney function, albuminuria–creatinine ratio was not available and renal function was estimated using the CKD-EPI equation, which has recognized limitations in obesity: creatinine reflects muscle mass, cystatin C is influenced by adiposity/inflammation; furthermore, BSA-indexing eGFR may introduce bias during weight loss when both body composition and BSA change simultaneously [33]. Finally, this study does not allow us to establish a definitive cut-off for pathological RSF accumulation. Further research is needed to determine the RSF threshold beyond which renal function is affected.

In conclusion, WHtR was associated with RSF and was the only anthropometric index independently associated with renal sinus fat percentage (RSF%). These findings support the use of WHtR as a simple, non-invasive surrogate marker for detecting individuals at risk of ectopic renal fat accumulation and its associated renal consequences. Future longitudinal studies with long follow-up are warranted to confirm the causal role of RSF% in kidney function decline and to determine whether interventions targeting central obesity can reduce RSF and improve renal outcomes. Furthermore, whether interventions that target central obesity, including weight loss, lifestyle modification can reduce RSF and translate into improved renal outcomes remains an important question for future research.

## Data Availability

Some or all datasets generated during and/or analyzed during the current study are not publicly available but are available from the corresponding author on reasonable request.
